# A Prize Essay on Anæsthetics

**Published:** 1864-05

**Authors:** 


### A PRIZE ESSAY ON ANÆSTHETICS.

The following resolution was offered and passed by the Mississippi Valley Dental Association. We direct the attention of our readers to it:

1—*Resolved*, That a gold medal, not exceeding $100 in value, be awarded by the Association for the best Essay on Anæsthetics, to be approved by a committee of the Association.

2—That essays competing for prize must be placed in the hands of the committee as early as January 1st, 1865.

3d—That the committee have power to reject all essays presented, if regarded as unworthy of the award.

4—That rejected essays be promptly disposed of by the committee as directed by the authors.

5—That when the committee has made the award, the approved essay be sealed up, and thus preserved till the committee report to the Association, when it and the envelope containing the author's name are to be opened.

6—That the copy-right shall belong to the Association.

7—That the committee shall not permit any one to inspect or see the copy of any essay till after making a report to the Association.

8—That the committee report at the next annual meeting.

The following persons compose the committee to report upon the Essays presented:

J. TAFT, JAS. TAYLOR,
GEO. WAIT, A. BERRY,
GEO. F. FOOTE, Cincinnati.

